# The Experiences of Hispanic Mothers of Young Children in Accessing Cross-sector Federal Financial Assistance Programs to Improve Family Self-sufficiency and Well-being

**DOI:** 10.1007/s40615-025-02493-8

**Published:** 2025-05-29

**Authors:** Vanessa Thorrington, Elizabeth Howe, Elizabeth Campos, Rebecca Bulotsky-Shearer, Ruby Natale, Imelda Moise

**Affiliations:** 1 Department of Psychology, University of Miami, Miami, FL USA; 2 Department of Pediatrics, University of Miami, Miami, FL USA; 3 Department of Geography and Sustainable Development, University of Miami, Miami, FL USA

**Keywords:** Early care and education, Mothers, Hispanic, Young children, SNAP, TANF, Housing assistance

## Abstract

This study explored the experiences of Hispanic families accessing subsidized early care and education programs, and their access to other federal financial assistance programs, designed to increase family self-sufficiency and well-being. Federal programs include subsidized early care and education, Supplemental Nutrition Assistance Program (SNAP), Temporary Assistance for Needy Families (TANF), and Housing and Urban Development assistance programs. Understanding Hispanic families’ perspective of barriers faced when accessing federal financial assistance programs is vital for policy changes to improve accessibility for these underserved families. The purpose of this study was to explore the voices of Hispanic mothers about their experiences accessing cross-sector federal financial assistance programs to gain an understanding about how these programs improve their self-sufficiency and family well-being. Qualitative methods were used by conducting 10 interviews with Hispanic mothers of young children under 5. Interviews were conducted in the preferred language of the mothers (English or Spanish) by trained staff. Interviews were transcribed and coded by four trained coders to achieve reliability. Data from the interviews were analyzed using grounded theory to code responses and develop meaning about mothers’ experiences. The findings illustrate the interplay between different types of cross-sector federal financial assistance programs that support families and how they promote the education and health of young Hispanic children and their families. The findings can be used to guide policy decision-making and future research.

## Introduction

### Federally Funded Early Childhood Care and Education Programs

Early childhood care and education programs (ECCE) are a vital social determinant of health. Extensive research documents the impact on young children’s development, school readiness, and longitudinal academic outcomes [1, 2]. Affordable ECCE programs assist families in meeting their child’s developmental needs, as well as enabling parents to work [3, 4]. Enrollment in ECCE programs is costly and places a significant burden on low-income families who may struggle with housing and food insecurity [5, 6]. Federally funded ECCE programs, such as Head Start (HS) and Early Head Start (EHS) and federal child care subsidy programs funded through the Child care and Development Fund (CCDF), can cover or offset the high cost of child care.

Federally funded ECCE programs address the needs of low-income families by connecting them to a multitude of services, including child care, with the objective of increasing family well-being and self-sufficiency and reducing the number of families living in poverty [7]. These programs allow families to access stable ECCE, which can reduce employee absenteeism by covering the full or partial cost of enrollment [7, 8]. Additionally, given federal and state regulations governing free and subsidized ECCE programs, when using these programs, there is a greater likelihood that a child will be enrolled in a licensed child care program or program meeting standards of state quality improvement systems [8, 9]. However, accessing ECCE programs is challenging, especially for Hispanic families [10–14] including those from immigrant backgrounds [15, 16].

Many studies have sought to understand how Hispanic mothers from mixed immigrant status families (a family whose members include people with different immigration statuses) [17] find and enroll their children into ECCE programs [3–6, 12, 18]. Hispanic families choose ECCE programs for many reasons including the quality of the program, affordability, availability, referral sources (including knowing someone whose family member attended the program), the type of setting (i.e., center-based versus family care), location of the program, need for full-time versus part-time care, support for their child’s special learning needs, or to ensure their child is ready for Kindergarten [18–21].

Despite the importance and need for ECCE programs, there are multiple barriers for Hispanic families searching to enroll their young children, including a family’s immigration status which further magnifies existing systemic barriers [2, 15, 22, 23]. For example, Hispanic families are twice as likely to live in low-income communities with fewer ECCE programs compared to non-Hispanic families [11, 14, 24, 25]. Barriers for Hispanic families when searching for and enrolling their children in an ECCE program include cost/affordability, location, lack of transportation, hours, bureaucratic complexity, and mistrust of government programs [10, 13, 26, 27]. Ongoing systematic and infrastructure challenges nationwide in the ECCE industry create additional barriers for families, reducing available slots in programs, particularly for infants and toddlers [28]. Parent reports of difficulty finding ECCE and disruptions in care have shown a long-term negative impact on child well-being [29]. Additionally, the pandemic further worsened industry challenges, with many teachers not returning to or entering the ECCE workforce [29–31].

### Cross-sector Federal Financial Assistance Programs

Families of young children who receive Supplemental Nutrition Assistance Program (SNAP) and/or Temporary Assistance for Needy Families (TANF) benefits are categorically eligible for enrollment in HS and EHS [32]. In addition, the U.S. Department of Agriculture issued guidance in 2018 for how the federally funded programs, HS and EHS, CCDF and the Special Supplemental Nutrition Program for Women, Infants, and Children (WIC), could collaborate to enhance families’ experiences by improving program care coordination and service delivery [33]. The purpose of this federal guidance was to minimize the burden on families by coordinating assistance programs so families could more easily participate in services for which they are eligible. Many barriers to access remain, despite the array of these federal financial assistance programs available to low-income families of young children.

### Study Purpose

The purpose of this study was to gain an understanding from Hispanic families’ about their experiences with cross-sector federal financial assistance programs intended to promote family self-sufficiency and well-being, including the enrollment process and challenges, as well as benefits from participation. While there has been considerable research on families’ experiences accessing ECCE programs, much of the existing findings are primarily quantitative relying on secondary data analyses of the National Survey of Early Care and Education [19, 20, 27, 34, 35], limiting a more detailed understanding of mothers’ experiences. Moreover, little information is available about families’ experiences in accessing multiple cross-sector federal assistance programs (i.e., SNAP, TANF, housing assistance) in addition to ECCE, therefore limiting knowledge about how families utilize multiple financial assistance programs, the benefits they experience, and the challenges they encounter. To address these gaps in the field, the present study uses a qualitative approach to obtain, in the words of Hispanic mothers, their experiences accessing cross-sector federal programs, such as ECCE, SNAP, TANF, and public housing assistance. Qualitative research methodology captures Hispanic mothers’ voices, allowing a more nuanced understanding of their experiences, which can guide future research and policy-making decisions.

This study used qualitative methods to gather information about families with young children’s patterns of service usage. The services of interest include access to ECCE, particularly their usage of HS and EHS and CCDF subsidized child care programs, SNAP, TANF, and housing assistance. The following research questions guided the methods.

1. Why do families choose their ECCE arrangement?
2. How do families choose ECCE and other cross-sector federal financial assistance programs?
3. How do families perceive the utility and quality for ECCE resources and other cross-sector federal financial assistance programs?
4. What are the barriers to accessing ECCE resources and other cross-sector federal financial assistance programs?

ECCE resources include finding and enrolling in free and subsidized programs to offset the cost of child care. Cross-sector federal financial assistance programs include families’ use of SNAP, TANF, and public housing assistance. Recruitment for the study began following approval from and following all ethical procedures of the authors’ Institutional Review Board.

## Method

### Participants and Setting

Interviews were conducted with 10 mothers living in a primarily Hispanic neighborhood in a suburb of a metropolitan area in the Southeastern United States. The neighborhood is composed of Hispanic immigrants and first-generation Hispanic Americans. The older population is mainly of Cuban ancestry, with a growing number of residents from Central and South American origin. Families living in this community have low to moderate income and are disproportionately negatively affected by social determinants of health, such as housing and food insecurity, and lack of access to child care. Purposeful sampling methods [36] were used to recruit Hispanic families with different experiences accessing ECCE and other cross-sector federal financial assistance programs to identify families with different patterns of service usage. Demographic information about both children and mothers can be found in Table 1.

**Table 1:** Child and mother demographics

Demographics (*N* = 10 mothers)	*N*
Family size	3.3
Children’s age	
2 years or less	8
Between 2 and 3 years	2
Children’s gender	
Male	4
Female	6
Children’s ethnicity	
Hispanic	10
Non-Hispanic	0
Children’s race	
White	5
Black	0
Mixed	2
Unknown	3
Mother’s age	
20–29	5
30–39	5
Mother’s ethnicity	
Hispanic	10
Non-Hispanic	0
Mother’s race	
White	5
Black	1
Mixed	1
Unknown	3
Mother’s educational level	
High school degree/some high school	3
College degree/some college*	7
Mother’s employment status	
Yes	8
No	2

^*^Some families indicated they obtained their degree in their home country

### Recruitment

Families were recruited through community-based ECCE centers serving families in the community. The research team began by identifying interested ECCE program directors who were contacted by phone to ask for their help in recruiting families from their centers. Research assistants and doctoral students facilitated recruitment by visiting these ECCE centers and asking mothers if they would like to participate. Families who expressed an interest in participating in the interviews were then contacted by the research team via text messaging to identify days and times for the interview. All families were compensated with a 100-dollar gift card.

### Procedures

The interviews were conducted using a semi-structured interview guide [37]. The interview guide was developed by reviewing websites of federal- and state-level governmental agencies for child care and other cross-sector federal financial assistance programs. In addition, a report detailing interviews with administrators of county/state ECCE programs, SNAP, and TANF was used to inform question development [38]. Drafts of the interview questions were reviewed and refined iteratively by the research team, including feedback from the members of county agencies and community-based organizations. The interview guide included main questions for each domain of interest along with multiple probe options to elicit more information from the interviewees [37]. The questions for the interviews can be found in Table 2.

**Table 2:** Interview questions

Domain	Interview questions
**ECCE**	
Family Choice	During the week, when your child is not with you, what is your childcare arrangement?
	Thinking back to when you first started looking for childcare, what were you looking for?
	What was most helpful to you in finding your current childcare?
	How did you find your current childcare center?
	What made you decide to enroll?
	Once you found the center, how long did it take to enroll your child?
Family Perceptions about Care	How has your experience been with your childcare program?
	Is there anything you don’t like about your childcare center?
Free or Subsidized ECCE	Are there any programs that help or have helped you pay for your child’s care?
	How did you find out about [funding source]?
	What was the process like to sign up for [funding source]?
	About how long did the entire application process take for you?
	What parts of the application process were easy? What parts of the process were difficult?
	What changes would you recommend to make the application process easier?
	Once you were accepted into the program, what were the next steps?
**Cross-Sector Federal Financial Assistance Programs**	
Finding Programs	Do you now, or have you had, experience with SNAP, TANF, or Housing benefits?
	How did you learn about [service] benefits?
	Did anyone explain the process to you?
Enrolling in Programs	What was the process to sign up for [service]?
	Would you say the overall process was easy and simple or difficult and complicated? Why?
	Have you ever reapplied for [service]? What was that process like?
Family Perception with Programs	How have [these service/benefits] helped your family?
	Would you access these services again if needed? Why or why not?

Cognitive interviews were conducted virtually with two mothers of young children affiliated with the county agencies, using Zoom Video Communications, to further refine the interview to ensure the questions would provide the data required to answer the research questions. The interviews with families were conducted by the research team, which included a combination of a doctoral-level university researcher, research assistants, and doctoral students fluent in Spanish. The research team received didactic training from an expert on qualitative research for conducting interviews prior to data collection.

The interviews were conducted virtually using Zoom Video Communications. Consent to participate from mothers was obtained verbally prior to the start of the interview. All interviews were recorded, and recordings were uploaded to a secure cloud-based storage, then saved and de-identified using a unique code to allow for anonymous transcription. Initial transcription was obtained using Zoom technology. Next, each transcript was reviewed using the recording, then amended and cleaned by the team member who conducted the interview. Cleaned transcripts were translated into English by the original interviewer and then back translated into Spanish by team members certified as fluent translators. Interviewers and back translators collaborated on the final transcripts, making notes regarding their choices. All translated transcripts were stored in a secure web-based cloud storage system for analysis.

### Data Analysis

The analysis was conducted by members of the research team trained in qualitative methods. All coding was conducted first in Microsoft Word using the comments feature. Coder’s files were merged to compare codes and ensure reliability. Due to the number of interviewers/coders, 60% of the transcripts were double coded at the first level of open coding, described below. The interrater coder reliability using the Kappa coefficient ranged between 0.94 and 1.0. This Kappa was computed for six separate interviews using transcripts coded by three different interviewers, each in comparison to a lead researcher. The high reliability can be attributed to a reiterative training process. The first set of interviews were reviewed by the lead researcher/trainer and each interview/coder before the interviewer/coder proceeded to code a second interview. The participant’s response to each question or prompt served as the coding unit for reliability. For the six interviews used to determine reliability, the interviews contained between 18 and 53 code units [39]. To confirm reliability in open coding, both coders needed to have coded the same elements and content of the participant’s response without inferring or changing meaning, as “language plays a crucial role in how and what we code” [40].

The first step in the coding process was to open code the transcripts using grounded theory [41]. During open coding, coders reviewed the transcripts creating a code where meaning could be chunked. These codes used as many of the participants’ words as possible without making conclusions or inferences. Additionally, during this step of the process, the coders selected quotes directly from the interviews to identify pertinent quotes “serving as symbolic markers of participants’ speech and meanings” [40]. In the next step, the analysis team used focused or axial coding to reduce the data by connecting statements with similar meanings. This was done by extracting the codes from Microsoft Word and pasting them in an Excel spreadsheet where they could be compared and collapsed. Open codes were placed on excel spreadsheets by topic of question and or prompt that the code answered; examples included “activities with family,” “finding child care,” and “application for federal financial assistance programs”. For each topic, researchers conferred to create a list of axial codes or categories [40]. Three columns were created to the right of the open code, as more than one of the created axial codes could apply to the open code. For example, axial codes were developed for the question asking mothers what was important to them when looking for child care providers: “social worker” (to designate when a family had used a social worker), “safety,” “child comfort,” “recommendation,” “physical location/features of a building,” “proximity to home,” and “child education.” Finally, themes were created by gathering codes in each category which revealed relationships, processes, and patterns. For all levels of coding, the team used discussions around agreements and disagreements, and constant comparisons to build consensus around the meaning of the statements from the interviews [42].

To validate the findings, the team relied on member checking with the participants. Participants were asked at the conclusion of the interview if they were amenable to a follow-up call from the interviewer to share findings. Of the full sample (*N* = 10), eight families (80%) reviewed the transcript from their interview and confirmed the accuracy of their transcript. Participants who agreed received an additional gift card for reviewing the findings. In addition to member checking, the results from the interviews were compared to earlier interviews with early learning and federal financial assistance program administrators.

## Results

Five overarching themes were generated, three related to ECCE and two related to federal financial assistance programs. For ECCE programs, major themes include finding and enrolling in a program, the application process, and mothers’ experiences. For cross-sector federal financial assistance programs, primary themes include application process and challenges and mother’s experiences.

### ECCE Programs

Eight of the 10 participants received financial assistance to pay for their ECCE program. Of these eight, the majority (*n* = 7) were enrolled in an EHS Program; one was using the Children’s Trust Thrive by 5 Families Forward scholarship program, a county-subsidized child care program federal CCDF child care subsidy program. Participants shared that they learned about their ECCE program from family, friends, neighbors, and social workers. Those participants who worked with a social worker to find their ECCE program reported fewer challenges with the process of finding and obtaining care. One mother explained how the social worker was helpful, “Honestly, she was really kind and did all the work basically. She explained [sic] me step by step how and gave me the flexibility to send the documentation by mail”. Participants’ experiences with their ECCE program showed consistency in satisfaction, adaptation, and child learning. See Table 3 for themes and quotes.

**Table 3:** Themes, sub-themes, and participant quotes

Theme	Sub-themes	Exemplar quote(s)*
Factors in Finding and Enrolling in ECCE		
	Referral Sources	“Through referral. So one of my best friends takes their son there. So I went ahead and, you know, looked into it.”
		“Well, this center had been recommended to me and I had heard about it from other moms”
		“Because they spoke very well about this center. Because a friend has her, her baby and she told me that it is a very good center: that the children have a good learning, according to her, she saw a progress in her son, that they took great care of him, they put him to do different activities to help them develop. That’s what motivated me the most to apply to this center”
	Safety	“First, it was a place where they looked, first, very safe. The center to be safe that it had, that is not. There are many centers that are near the street that you see the child in the field is very near the street. Then, for us, that was not safe because in fact we have seen cases and news of people that suddenly enter and rob the kids or they start talking to them, or they give them sweets, you see, and that is very dangerous.”
		“Well, I was looking mostly for a place where I can feel, you know, today we see so many things like child abuse that, like, they are helpless creatures. Sometimes, when one sees cases, being honest I was, I spent almost 2 years without working due to that same fear. Well, here, us the Latinos, or well I’m, in my case, I’m alone. Like, we are alone. We don’t have much family so, I don’t have someone that I trust to take care of her. So, like, I was looking for a place in which I could feel safe and peace when leaving he there more than anything.”
	Location	“Like, yes, the location also influenced. It’s something near where I lived, live, my jobs and also that is a place where I can get either by public transportation, is very busy area, transportation.”
		“Oh, yes, honestly, it has a very very good location. It is convenient for me because it is near my school it’s on my way.”
	Staff Professionalism	“What helped me decide was that, that when I went to both day cares I saw that it was much more professional the teamwork that they have at the day care, and the installations and how they maintain it, clean.”
		“That all the people and staff and also the installations were clean, organized and the people the teachers looked professional because you do not know, you do not know, if they were professionals from the first glance, I mean, after getting to know them. Then I was going to realize. That at least they looked like responsible and professional people, and that the child felt good.”
	Overlap with Federal Assistance Benefits	“To apply to the scholarship I had to have the food stamps. So, I applied to the food stamp. Once they gave me the food stamps, I sent the information. I sent the food stamp’s information, the WIC, and the vaccines up to date. Then, in that moment, I was able to apply to the scholarship for the day care.”
	Challenges	“Not because there was quota or because a child left but because they opened a new classroom and that was when they could, like, enroll my daughter. And I had to wait just like 2 weeks while they arranged the classroom with all the safety measures.”
ECCE Application Process		
	Online vs. In-Person Application	“I feel that they should give the option again [in person] for the people that are not very familiarized with technology or being able to send emails and let the parent go and do it like I did it at the beginning.”
	Community Support	“I searched it up in the internet, the person also, the social worker, I went to visit her, and she gave me a list of centers. She gave me a list of centers and I applied.”
		“Before presenting myself at the daycare to meet the director and get to know the building where she was going to be, the social worker told me that the papers that I had to take, the steps that I needed to do, it was fast and I didn’t have any issues.”
	Wait Time	“So, I wish, I don't know, that they would help a little more, that the process was a little more I don't know, like more, that it didn’t take so long.”
		“They called me to tell me that first, because they placed me in a waitlist and then they called me and told me that they had a place for my daughter, that I could take her. But more or less 2 or 3 months passed.”
	Application Process & Challenges	“I called and said that I wanted to apply, that I was a single mother, that they gave me that number. So at that time, they told me that there was no quota [capacity] but to send the girl’s information, her birth certificate, to send her vaccines, the food stamps, WIC and that when they had quota, they would reach out.”
		“But I had to have sent some information, but I didn’t send it, I didn’t know and the email with the information they were asking hadn’t arrived and no and so, no, I lost [the application]. I didn’t do it. I lost it. They closed it for missing information.”
		“They asked like information about who lives in your house, about your if you don’t make certain amount of money to apply to the scholarship, because that something for people like from low resources that really need it. they asked information, the girl’s birth certificate, and I left the application. after that, I waited a year. And it was when a worker, I think the social worker that works directly with the day care a woman called [*name of social worker],* and from there she was the one that assisted me.. Because, as with all the applications, I did it when the girl was born. She had the vaccines expired and it was other kind of paperwork. She called me and helped me to finish the process, or to start the process again.”
	Mother Recommendations	“There, do the written application at the center directly.”
		“Well, I believe that there is not much to do because for a child to be accepted, another one has to leave. There is not much to do. Unless the day care was bigger, there would be more classrooms and they could accept more children.”
Mother’s Experiences with their ECCE Program		
	Satisfaction	“They educate them in the best way, teach them. It teaches children independently to create very important habits that are going to be very important not only now but also in school and in life. And they have good childcare, very respectful. For me it’s the best.”
	Child Comfort/Adaptation	“..he adapted very well because he adapted better that I thought, eh didn’t cry- the first 2 days [he did] but that was it, he didn’t cry anymore. He goes in by himself. He goes with her, stretches his hand, he goes and doesn’t cry.”
	Child Development	“She talks more. She has learned a lot of words. For example, she already knows how to count from 1 to 10. She has learned the names of all of the children in her classroom, emm, she has motivation to paint and is more sociable and independent.”
	Children with Developmental Delays or Disabilities	”Yes, he has developed a lot. My child has a little delay in his speech and thanks to the teachers and therapy and things they give him he has advanced a lot. He speaks more, knows how to express himself better when he wants something, before he used to not do that, he would only cry. And thanks to that school and the education they offer I have seen a lot of change and a lot of development in the child, yes.”
Cross-Sector Federal Financial Assistance Application Process and Challenges		
	Referral Source & Assistance	“Someone helped me with the food stamp application. Someone my friend knows that also helped her with that.”
		“When I got pregnant, a friend spoke to me about the benefits and Medicaid, and she helped me, I mean, guided me and explained how the process was, and I applied.”
		“I found out about the Food Stamp when I, I am about to turn 8 years in this country. I came and I was pregnant with the eldest child. So, as I was single, I came with a family member and that family member was the one that told me about the Food Stamps.”
	Communication	“Now like, they have the computers, literally there are the computers. They tell you like that ‘sit there, bring the user number, password and do it. And follow the steps that you need to do’ that’s how they tell you.”
	Personnel	“But then, I understood how to do everything and for me is easier doing it online because if one goes straight where they help you, people there are very, like, they are very rude, they don’t help, don’t explain things properly, they say thing in a reluctant manner.”
	Scheduling	“Well, in first place, the complicated was that I went to the office to do the line at around 5 in the morning because they told me that I had to go early to do the line. I went to do the line. I went and at 8 in the morning they started to attend. And when they attended me, they told me, “we don’t attend, just with an appointment. You have to give a code and make an appointment. Once you have your appointment you can come here and starting from 8 in the morning. Then, yes, I made the appointment like that, through the code. Then they gave me the appointment, I was able to go to the office and do the process”
	Documentation	“But it was my fault because I didn’t send the documents as I should, I didn’t send all the documents. So, then, I called them and they explained to me “you are missing this and this. You have to send it to this fax number.” Then, I did everything like they told me. I sent everything and the next day I had the benefits.”
	Wait Time	“Well, it’s similar to the first time. The same way, one makes an appointment, goes and talks with a worker, they give and send you the information and tell you to wait 30–40 days for a response.”
		“They give you a month of wait to receive a respond about the process.”
		“I just uploaded, fill out the information about the establishment, make and attached the documents that they asked and like 2 months later they gave me an answer. 2 or 3 months more or less.”
	Reapplication	“So, if I didn’t had the option of doing it online, I think it would be 100% difficult to be able to renew my daughter’s food stamp.”
Mothers’ Experiences with Programs		
	Usage & Direct Benefits to Families	“So, now that I’m without him [father], like, it’s been a really huge help for me because I can that, like, I get more money to cover and be able to pay the rent and other bills and so do my budget with the SNAP a little more to be able to feed.”
		“They have helped a lot, because realistically the cost of living has increased a lot here, everything has increased.”
		“Well, a lot. For example, with the SNAP and WIC I can buy everything that the girl needs.”
		“It helped me a lot to be honest. Because, well, I work at a restaurant and sometimes, yeah sometimes they don’t give enough because they pay by hour. And honestly with what I make, honestly, It’s not enough. It’s worst with the rent that now it’s super high honestly. So, with the food stamps it really helps me a lot being honest.”

^*^The quotes presented in this table are exemplar quotes and not all quotes from the data

#### Factors Finding and Enrolling in an ECCE Program

The most often cited factor when looking for an ECCE program among the participants was a referral source. Nine of the 10 participants noted they were referred to the center in which they ultimately enrolled. The referral sources mentioned included friends, neighbors, relatives, and EHS social workers. Participation in other federally funded assistance programs, such as SNAP and WIC, were also a factor in enrollment in EHS. The second most often cited factor used in looking for an ECCE program was the center’s location. Some mothers noted using Google to find the closest center, while in other cases, the identification of the closest center was facilitated by EHS personnel. Safety and child comfort were also identified by more than half of the participants as important factors in their search for child care.

One mother commented on features of the building as contributing to safety, “They told me that it was something very safe, that they were super strict…the maintenance of the day care, the teachers, the person who administers the place was super responsible.”

Participants noted some challenges they faced in enrolling their child in their ECCE program, including a lack of available slots in a preferred center. See Table 3 for additional exemplar quotes related to the theme, “Factors finding and enrolling in ECCE.”

Once parents found an ECCE program, parents made the decision to enroll their child. Mothers’ decision-making factors for enrollment were similar to their initial search when looking to find a child care program. When making the decision to enroll their child, participants most noted the location of the ECCE program as an important factor. The next most common factor for enrollment was personal referrals parents received for the center they chose. When noting the importance of the staff at their center as a decision to enroll, participants articulated that the staff were “professional” and/or “trained.” Other themes emerging were that several participants toured the center before enrollment and the cleanliness of the center played a role in their choice.

“I liked, you know, the energy of the school. When we did the tour…I was happy with, you know the little library. They have set up all the rules, and you know, the day to day that the kids go through. And I sat back. I watched my son interact with the teachers and the and those other students. And I was like, satisfied with you know what I was seeing.”

“What helped me decide was that, that when I went to both day cares I saw that it was much more professional the teamwork that they have at the day care, and the installations and how they maintain it, clean.”

Timelines reported for enrollment ranged from 3 days to 6 months. Enrollment time is described by participants as the time from completion of paperwork at the ECCE program to the child starting to attend daily. The longer enrollment times, 3 and 6 months, involved issues with capacity. These ECCE programs were at capacity, and mothers needed to wait for a spot to open for their child or for additional funding to the program to start a new class. The majority of participants’ children were able to start at their center between 3 and 5 days of completing the enrollment paperwork.

#### ECCE Application Process: Ease and Challenges

Participants reported that the sign-up process for their ECCE program was an online application (aligned with the procedures for child care enrollment in the state in which this study was conducted). Most participants reported that it was an easy process. This process was supported in some cases by the help of a neighbor, relative, or social worker. Challenges to the process included variations of waiting, mostly for responses; also one mother waited for a classroom to be set up for her child to start.

“Mothers, we have to work, sometimes we can’t leave him (her child) with just anyone, because you can’t leave him with anyone, and so it's hard to see and so to leave a child in a places is like trusting in that person because you aren’t leaving your wallet or leaving your card or phone, you're leaving something that is yours, that you are going to trust in this person to watch, take care of them for you. So, I wish, I don't know, that they would help a little more, that the process was a little more I don't know, like more, that it didn’t take so long.”

Reported challenges include applications being closed and wait times. Reasons for application closures were due to missing or incomplete paperwork. Another participant was directed to an ECCE program farther from their house, as their first choice had no openings.

“When I got the option, and the social worker told me to accept it because as they [sic] already have given her the scholarship and that if I wanted to transfer her later it was going to be an easier process. So, I placed her there. Now she, when she sees the teachers that are there, runs. So, I feel more like, even though I have to go, like I’m telling you in Uber or go in a bus, but I feel that she is fine there.”

Application wait times ranged between 2 weeks and 1 year. The reported one year wait times included issues with documentation. Most participants waited between 2 weeks and 3 months for information regarding a scholarship program for their child.

When asked what recommendations participants had to improve the application process for ECCE programs, many noted that the option should still exist to complete the application in person. It was also noted that help should be provided with a written application at the center. For example, one mother stated,

“I feel that they should give the option again for the people that are not very familiarized with technology or being able to send emails and let the mother go and do it like I did it at the beginning.”

Another participant noted that there should be more help with the process overall. See Table 3 for exemplar quotes.

#### Mothers’ Experiences with Their ECCE Program

Throughout the interviews, mothers expressed satisfaction with their current ECCE program. They noted they felt their children were “taken care of” by their child’s ECCE providers. At least half of the participants reported they would send another child to the same center.

“For me personally, I love that center and I chose it very well. In fact, when they assigned it to me, one always looks for opinions that people have, and everyone had very good opinions. And I am one of them. After I had the experience of my child participating.”

Mothers also observed how their children adapted to the center in positive ways and particularly to new people. Mothers also described positive changes in their child’s development once they started attending their program. For example, mothers noted that their children increased their independence, especially in feeding since starting at their centers. Additionally, over half of participants reported advances in their children’s language development, as seen in their ability to express themselves. Additionally, some children are receiving therapy related to their speech and language development in the child care program. See Table 3 for exemplar quotes regarding parent experiences with child care programs.

### Cross-Sector Federal Financial Assistance Programs

While most participants noted the application process to access cross-sector federal financial assistance programs was “easy,” there was a consistent theme with those participants that did experience challenges. Additionally, mothers accessing these programs readily noted how these benefits had assisted their families and affirmed that they would access the programs for their families again.

#### Application Process and Challenges for Federal Financial Assistance Programs

Three of the participants noted challenges with the application to obtain services. While these challenges were varied, all involved issues with communication. One participant went early and in-person to the local service center for SNAP to get an appointment only to find out that scheduling appointments must be done on-line. Another participant reported complications with scheduling a phone interview and being asked to send additional documents. Lastly, a participant reported a lack of assistance when applying at the service center.

“Now like, they have the computers, literally there are the computers. They tell you like that sit there, bring the user number, password and do it. And follow the steps that you need to do’ that’s how they tell you.”

Others stated the process was “easy” and attributed this response to their understanding of on-line processes, ability to upload documents, and/or assistance from friends or social workers. Participants observed that the process has changed and that their ease could be attributed to the previous process in which staff at the service centers assisted with the application, specifically by filling out surveys and submitting documents. Participants consistently reported a wait time of approximately a month to learn their benefit status for SNAP. One participant reported a 2- to 3-month wait. When they checked, they learned they were missing documents.

Most participants noted they had reapplied for services. When they reapplied, the process was similar to the initial application. One participant explained the process, “They asked me to see that I’m not earning more or earning less and as it’s almost similar what I’ve been earning. So, it gets renewed through the web page and then everything gets automatically updated.”

Participants’ recommendations to improve the process of applying for federal financial assistance centered on personnel and communication. Several participants noted that staff at local service centers exhibited a lack of professionalism and or training to assist applicants with the process. Participants also expressed concerns for those in their community that may not have the resources or abilities to provide documentation in the mode that was required. For example, one mother noted,

“But imagine that-that someone that a disable [sic] person goes, to say it in a way, and like they don’t know, they say that they can’t help you then, how is that person going to be able to do it? Or for example, someone that doesn’t have a stable mean of transportation, you have to go to the library or to some place where they do copies and they you have to go back to the place.”

#### Mothers’ Experiences with Federal Financial Assistance Programs

Eight of 10 participants responded “yes” when asked about the use of federal financial assistance such as the SNAP, TANF, or public housing. Seven participants responded yes to being enrolled to receive SNAP; two of these participants also noted being enrolled in WIC. Four participants noted enrollment in Medicaid by someone in their family. Seven participants named their referral to these services, two by a friend, two by a family member, two by a social worker, and one by the child care center their child attends.

#### Benefits to the Family from Participation in Federal Financial Assistance Programs

All the participants receiving assistance said that they would apply again if needed. Several participants noted they had reapplied previously, as it was necessary to keep the benefits continuing. However, other participants had allowed their benefits to lapse. One participant mentioned the cost of living as a factor in her need for assistance, another participant remarked she uses SNAP and WIC “to get what her daughter needs”. Other participants noted unique relationship status and work situations were factors in the importance of the aid for their families. One mother noted how much the assistance had benefited her family, “Greatly, because honestly, like I explained, I study, my husband is who works. And then that helps greatly to eat regarding nourishment, mainly for the children.”

## Discussion

### ECCE Programs

#### Finding and Enrolling in ECCE Programs

Referral sources, safety of their child, program location, staff professionalism, overlap with other federal financial assistance, and challenges encountered all emerged as important aspects of finding and enrolling in ECCE programs for these Hispanic mothers. Referral sources included family, friends, and EHS social workers. The importance of the referral sources illustrates the importance of trusted community references for selecting a program for their child [4, 6, 21]. The mothers interviewed, especially those in households of mixed immigrant status, relied on a familiar or trusted community referral source to ensure their children’s safety in the program, as is consistent with prior research findings [3, 5, 6, 12, 18, 21, 43]. The physical environment, the professionalism of staff, and the perceived safety of the program all contributed to mothers’ decision-making when choosing and enrolling their child. This finding aligns with prior research indicating that these elements of the program are influential for mothers, likely contributing to a sense of comfort about leaving their child in others care [43].Visiting the center with their child contributed to mothers’ comfort by allowing them to witness interactions between their child and staff and observe the quality of the program environment and the professionalism of the staff. After enrollment, visits to the center gave mothers the opportunity to observe the happiness of their child, and other children in the program, such as a lack of children crying, and later contributing to a sense of satisfaction with the program.

#### ECCE Application Process and Challenges

Mothers described several aspects of the application process, including challenges such as applying online versus in-person, having support from a community member in completing the application, managing documentation, and wait times until enrollment. Mothers’ were also asked to provide suggestions about how programs can improve their experiences applying for and enrolling in ECCE. Not completing the application in its entirety or missing paperwork was noted as a challenge when applying for free or subsidized ECCE. Support from a community member, such as an EHS social worker, was a resource that helped mothers in dealing with these challenges. The empirical literature indicates that systemic and bureaucratic processes are barriers for mothers from low-income communities in accessing ECCE programs [10, 13, 26–28]. Addressing these barriers can increase enrollment, reaching a wider swath of Hispanic families with young children.

#### Mothers’ Experiences with ECCE Programs

Mothers’ reported experiences with their ECCE program illustrates the value they place on their child’s program and their overall satisfaction once their child is enrolled. Mothers felt their child adapted well to the center, including to become familiar with new people, and were well cared for by the center staff. Additionally, they reported observing that their child’s enrollment promoted development in positive ways, especially language acquisition and independence in caring for themselves. This finding highlights the role ECCE programs play in advancing young children’s development [14, 44, 45]. The findings align with prior research findings that document the positive effects of enrollment in ECCE programs on young Hispanic children’s development and school readiness [14].

Some of the mothers (*n* = 2) in this study reported their child receiving early intervention services in the classroom. Children with special needs spend many hours during the day in the ECCE classroom where they receive therapeutic services, which can contribute to their participation and engagement [46]. The participation of young children with disabilities in ECCE programs has long been cited as a mechanism for effective inclusion in classrooms with typically developing peers [47].

Moreover, the mothers in this study described children’s developmental accomplishments spilling over into the home and family routine, contributing to families’ feelings of well-being. Research indicates family well-being is critical for young children’s development [48, 49]. These findings highlight the importance of enrollment in formal ECCE programs for supporting and promoting positive outcomes in young Hispanic children.

### Cross-Sector Federal Financial Assistance Programs

Themes regarding mothers’ receipt of federal financial assistance programs included the challenges in the application process and their positive experiences with benefits once enrolled in the program. Most Hispanic mothers in this sample received at least one type of federal financial assistance. Participation in federal financial assistance programs can improve the health and well-being of Hispanic families from low-income communities. Despite the benefits of participation, research indicates lower enrollment of Hispanic families in these programs, which may be due to systemic barriers [50, 51]. In this study, mothers described using SNAP but not having experiences with other types of cross-sector federal programs such as TANF and housing assistance.

#### Federal Financial Assistance Programs Application Process and Challenges

Mothers who qualify for SNAP and TANF are categorically eligible for HS and EHS services [32]. This policy allows for better coordination between federally funded financial assistance programs. Better coordination between systems potentially can contribute to decreases in disparities in cross-sector service usage leading to improved developmental outcomes for young Hispanic children. Improved coordination can also eliminate barriers to finding and applying for publicly funded ECCE programs [7, 52]. None of the mothers in this sample reported using TANF, either currently or in the past. This finding corresponds with national data suggesting, despite being eligible for HS and EHS when enrolled in TANF, Hispanic families do not utilize this federal assistance program [52].

#### Mothers’ Experiences with Federal Financial Assistance Programs

Mothers’ uniformly reported satisfaction with receipt of federal financial assistance programs. The benefits to their family included offsetting the cost of living, such as housing and the cost of food. This finding aligns with prior research indicating that receipt of benefits supports family well-being and self-sufficiency is important for the health and education of young children and families, especially those in traditionally underserved communities [50, 51].

### Limitations and Future Directions

Limitations of this study include that the findings are limited to a small sample of Hispanic mothers, of whom some were from immigrant backgrounds living in an urban area in the Southeastern USA. Additionally, Hispanic families in the USA are heterogeneous, including their countries of origin, cultural variations, and differences based on state and local place-based contexts [53]. Secondly, the data in this study came from a majority (*n* = 8) of mothers whose children were enrolled in a single large-center-based ECCE program with primarily Spanish-speaking teachers. Moreover, this center participates in the county’s Quality Improvement System; therefore, experiences may differ from mothers whose children are from other types of centers who are not participating in quality improvement efforts.

Future research direction includes how our study findings advance the field by adding Hispanic mothers’ experiences and perspectives to the mostly quantitative body research on access to ECCE and federal financial assistance programs [34]. While nationally representative secondary data is helpful for obtaining a high-level understanding of mothers’ experiences, it does not provide more nuanced information that can be helpful for guiding policy changes at a local level. Given that most federal financial assistance programs are implemented and regulated through policies and guidance at a state or local level [54], having more nuanced information is critical.

Additional future directions include gaining a better understanding from families about the importance of safety when selecting and enrolling in child care programs. Mothers in this study reported that a necessary feature of their ECCE program was their perception of the safety of the center. Prior research indicates that low-income families are more concerned about safety than families with higher incomes [22, 55]. Concerns about ECCE program safety may be due to lower-income families’ exposure to neighborhood or community risk factors such as higher crime and incidences of violence [35]. Future research is needed to better understand the aspect of safety in order to meet family needs.

Some of the mothers in this study reported that the enrollment of their child with a developmental delay or disability in their ECCE program was positively associated with gains in development. There is limited understanding regarding the benefits of enrollment in child care programs for children with disabilities [35], making this an important topic for future research. Children with identified or suspected disabilities are likely to benefit from participation in child care programs, given access to same-age peers and a developmentally appropriate curriculum [47].

Many of the mothers in this study reported relying on people they knew from their community as a referral source for locating their ECCE program. Community members included family members, friends, neighbors, and EHS social workers. This finding aligns with prior research; however, little is known about why mothers trust these information sources and the extent to which these sources have reliable information [35]. Given some of the mothers in the study were from mixed immigrant status households, including immigration status in future research will be critical to understand why community members are viewed as trusted referral sources and who these messengers are in future research.

This study illustrated the overlap between the use of varied federally funded assistance programs by Hispanic mothers of young children. However, we need a better understanding of missed opportunities in service usage to understand how coordination can improve access to financial assistance programs [56]. Understanding how service coordination improves access is even more important for Hispanic families who experience barriers to accessing these programs (14).

## Conclusion

This study sought to gain a greater understanding of the experiences of Hispanic mothers of young children in accessing ECCE and other cross-sector federal financial assistance programs. Mothers described variations in locating and enrolling in publicly funded ECCE programs. However, once their child was enrolled, mothers uniformly reported being very satisfied with their program, especially regarding their child’s development. Most (*n* = 8) of the mothers in this study also applied for and received at least one other type of cross-sector federally funded financial assistance program in addition to their child’s enrollment in EHS. These mothers uniformly reported that enrollment in these programs benefited them by offsetting costs increasing to their self-sufficiency and family well-being. This study contributes to the literature by providing detailed information from the voices of Hispanic mothers of young children under 5 living in a historically marginalized community. Mothers reported on their experiences in locating and enrolling in ECCE and other cross-sector federal financial assistance programs, including application process and challenges and their satisfaction once enrolled. The findings can be used to guide future research and policy-making to increase access to family strengthening services that promote the positive well-being of young Hispanic children and their families.

## Data Availability

The data that support the findings of this study are not available due to the University IRB confidentiality/privacy restrictions of the participant data. Interested researchers should contact the first author in the case that selected de-identified data could be made available from the authors upon reasonable request and with the permission of the University IRB.
